# Magnetic Particles: Their Applications from Sample Preparations to Biosensing Platforms

**DOI:** 10.3390/mi11030302

**Published:** 2020-03-13

**Authors:** Seong-Eun Kim, My Van Tieu, Sei Young Hwang, Min-Ho Lee

**Affiliations:** 1 Human IT Convergence Research Center, Korea Electronics Technology Institute, Gyeonggi-do 13509, Korea; sekim@keti.re.kr; 2 School of Integrative Engineering, Chung-Ang University, 84 Heukseok-ro, Seoul 06974, Korea; tmvantp0113@gmail.com (M.V.T.); forsylife@gmail.com (S.Y.H.)

**Keywords:** point-of-care, sample preparation, diagnostics, biosensor, magnetic nanoparticles

## Abstract

The growing interest in magnetic materials as a universal tool has been shown by an increasing number of scientific publications regarding magnetic materials and its various applications. Substantial progress has been recently made on the synthesis of magnetic iron oxide particles in terms of size, chemical composition, and surface chemistry. In addition, surface layers of polymers, silica, biomolecules, etc., on magnetic particles, can be modified to obtain affinity to target molecules. The developed magnetic iron oxide particles have been significantly utilized for diagnostic applications, such as sample preparations and biosensing platforms, leading to the selectivity and sensitivity against target molecules and the ease of use in the sensing systems. For the process of sample preparations, the magnetic particles do assist in target isolation from biological environments, having non-specific molecules and undesired molecules. Moreover, the magnetic particles can be easily applied for various methods of biosensing devices, such as optical, electrochemical, and magnetic phenomena-based methods, and also any methods combined with microfluidic systems. Here we review the utilization of magnetic materials in the isolation/preconcentration of various molecules and cells, and their use in various techniques for diagnostic biosensors that may greatly contribute to future innovation in point-of-care and high-throughput automation systems.

## 1. Introduction

Magnetic particles typically refer to the materials consisting of magnetite (Fe_3_O_4_) or maghemite (gamma-Fe_2_O_3_) ranging from sub-nano to micro-meters in size which respond to an external magnetic field [1]. Due to their unique magnetic property, they have great potentials in a variety of biological applications in their bare form or coated with surface coating materials and functional groups chosen for specific uses [2,3,4,5,6,7]. Isolation and separation of specific target molecules, including small drugs, deoxyribonucleic acid (DNA), proteins, and cells from biological media are necessary for bioscience and biomedical applications. However, biological environments known as highly complex matrixes (i.e., serum, urine, saliva, etc.) and also typical separation processes, e.g., the silica-based column method, are required to have a time-consuming procedure, and even traditional ones, e.g., the phenol/alcohol precipitation method, require toxic reagents for the extraction of DNA/(ribonucleic acid)RNA, proteins, etc. [8]. In recent developments, magnetic particles can improve the efficiency of target molecule separations without harmful reagents. Due to different compositions, sizes and magnetic properties, magnetic nanoparticles (MNPs) can be used in a variety of instruments and formats for biosensing with an enhancement of sensitivity and the stability [9,10,11]. Thus, many types of biosensors have been using surface-functionalized magnetic particles to recognize specific molecular targets with high sensitivity and selectivity. In the field of biomedical applications, a wide spectrum of advanced materials, including micro and nanoparticles, have been developed for sample preparation and biosensing platforms [12,13,14]. The use of magnetic particles has allowed the biomedical processes to be quick, simple, robust, and high-throughput systems. In this review, a number of cases of magnetic materials applied in isolation/pre-concentration of various target molecules and their use in recent biosensing platforms for biomedical applications will be given. It has been highly anticipated that these current developments in magnetic particle may contribute to future innovation in point-of-care and high-throughput systems to increase the chance of successful diagnostics and clinical treatments.

## 2. Sample Preparations Using Magnetic Particles

Magnetic particles are considered interesting materials that are widely used in many applications, such as separation, diagnostics, and therapeutics. Magnetic nanoparticles can easily be separated from complex matrices such as biological samples by using an external magnetic field. Thus, traditional separation processes are not required, such as filtration or centrifugation. Magnetic solid-phase extraction (MSPE) was proposed for the facile and fast sample preparation processes. The magnetic materials are generally directly dispersed in the sample solutions for the rapid extraction process because they can be readily recovered by a magnet. Typically, the MSPE material is composed of a magnetic core and the functionalized outer surface of the same particle to be collected by using a magnet and for the extraction of various target compounds from biological samples (i.e., serum, urine, saliva, etc.), respectively.

### 2.1. MP-Based Drug Extraction from Biological Samples

MSPE for drug molecule extraction from biological samples (blood plasma, urine, etc.) is one of the crucial application areas of magnetic nanoparticles. Carbon coated magnetic Fe_3_O_4_ nanoparticles were used for extracting pharmaceutical compounds, including carvedilol, losartan, and amlodipine besylate, from plasma samples [15]. Magnetic nanocomposites composed of Fe_3_O_4_ nanoparticles and polyaniline were developed and successfully separated benxodiazepine drugs, including nitrazepam and lorazepam, from human plasma and urine samples [16]. Multiwalled carbon nanotube coated Fe_3_O_4_ nanoparticles used for the efficient extraction of brucine (a neurotoxic alkaloid existing in the Nux-vomica tree) from human urine samples were also reported [17]. For the selective extraction of seven estrogens from human urine samples, magnetic Fe_3_O_4_ nanoparticles having a layer composed of 1,3,5-triformylbenzene (Tb) and benzidine (Bd) (Fe_3_O_4_@TbBd) were prepared and successfully applied [18]. Organic dendrimer-modified magnetic Fe_3_O_4_ nanoparticles for the extraction of rosuvastatin from human urine and blood plasma were reported [19]. The magnetic Fe_3_O_4_ nanoparticles were first synthesized by using FeCl_2_ and FeCl_3_. The surface of the synthesized magnetic Fe_3_O_4_ nanoparticles was conjugated with organic dendrimers containing ethylene diamine and methyl methacrylate, and then applied for efficient separation, achieving 61 mg g^-1^. In addition, magnetic nanoparticles having a molecularly imprinted polymeric (MIP) layer were successfully developed and utilized for the selective separation of 9-hydroxyrisperidone and risperidone from human urine samples [20]. Polydopamine(pDA)-coated magnetic Fe_3_O_4_ nanoparticles modified with multi-walled carbon nanotubes (MWCNTs) were prepared. Owing to the pDA, it results in the formation of a continuous coating layer on the substrate magnetic material via strong binding affinity of catechol functional groups. The prepared magnetic nanoparticles were successfully used for the extraction of antiepileptic drugs, including phenytoin, oxcarbazepine, and carbamazepine from human urine, plasma, and cerebrospinal fluid samples [21].

### 2.2. MP-Based DNA/RNA Extraction from Biological Samples

Magnetic particles have significant advantages in both ease of use and high purity when isolating nucleated cells and nucleotides directly from biological samples. Although the extracted genomic DNA (gDNA) are easily contaminated by various known enzyme inhibitors (phenol, urea, and salts) originating from the traditional organic extraction and/or biological samples, this approach concentrates and extracts gDNA and eliminates the influence of physiological metabolites from the biological samples that would otherwise inhibit enzyme activities for polymerase chain reaction (PCR). Shan et al. used carboxylated magnetic nanoparticles (CMNPs) to develop a PCR-ready gDNA extraction from urine and blood samples. CMNPs were first used for cell extractions and then absorbed gDNA from the lysates. Relative to traditional methods, the present procedure required less handling, no hazardous reagents (e.g., chloroform), and could be carried out in single microcentrifuge tube within 30 min. These methods, while simple, rapid, sensitive, and environmentally friendly, are suitable for routine laboratory use, but also hold promise for the construction of automated urine extraction systems for various diagnostic purposes [22,23,24].

### 2.3. MP-Based Protein/Peptide Isolation from Biological Samples

The use of magnetic particles is highly convenient and rapid for separations of proteins and peptide. Magnetic beads exhibit low non-specific binding of non-target molecules included in different samples. For certain samples, preclearing may be required to remove molecules having highly non-specific binding affinity. First, the sample can be mixed with magnetic nanoparticles not coated with the affinity ligand. For the case of immunomagnetic separation, magnetic nanoparticles have been coated with secondary antibody or with irrelevant antibodies. In addition, the non-specific binding can also be reduced by adding a non-ionic detergent either in the sample or in the washing buffers during the isolation of the target. In general, magnetic particles for protein/peptide separation can be used in two different methods. In the direct method, an appropriate affinity ligand is directly coupled to the magnetic particles exhibiting the affinity towards target compounds. These particles are added to the sample and target compounds then bind to them. In the indirect method, the free affinity ligand (in most cases an appropriate antibody) is added to the solution to enable the interaction with the target compound. The resulting complex is then captured by appropriate magnetic particles. In both methods, magnetic particles with isolated target compounds are magnetically separated, and then several washing steps are performed to the remove majority of contaminating compounds and particles. The target compounds are then usually eluted. In most cases, bound protein/peptide can be added to standard elution methods, such as the change of pH, change of ionic strength, and use of polarity reducing agent (e.g., dioxane or ethyleneglycol). Affinity elution (e.g., elution of glycoproteins from lectin coated magnetic beads by the addition of free sugar) may be both efficient and gentle. The following examples are for the protein/peptide magnetic separations (Table 1).

## 3. Biosensing Devices Using Magnetic Particles

**Figure 1:**
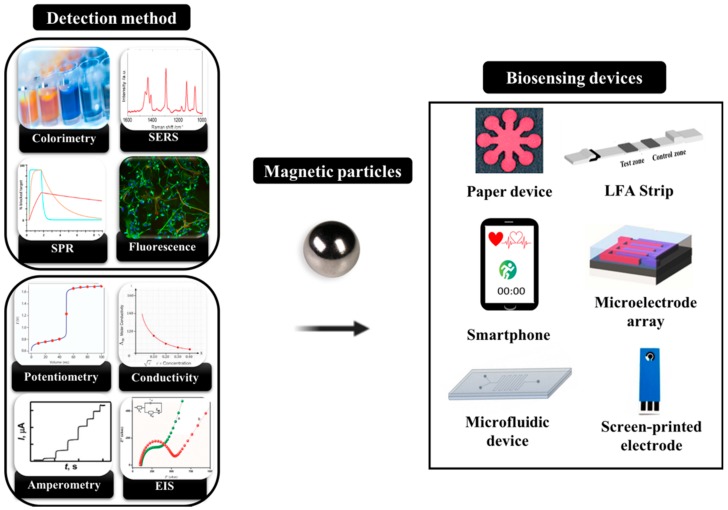
Schematic representation of the detection method based on either optical or electrochemical techniques combined with magnetic materials for biosensing.

In this section, we summarized recent studies of biosensing devices that apply magnetic materials. They have advantageous functional capabilities, such as their magnetic property, low background noise, good dispersions, and highly biocompatible surfaces with regard to the immobilized recognizable bioreceptors [34,35,36,37]. With the continuing multidisciplinary development of magnetic-particle-based biosensing techniques, these efforts have found the way for modern biological devices for on-site or high-throughput purposes by replacing sophisticated monitoring biomedical devices. Of the varied biosensing devices available, we have paid attention to two main biosensing devices; i.e., optical sensing devices and electrochemical sensing devices with the integration of magnetic material for sensing biological targets. As illustrated in Figure 1, we have paid much attention to smartphones and other mobile devices integrated with detection in paper-based analytical devices or lateral-flow immunochromatographic assays (LFIA)—and the interdigitated array microelectrode, screen-printed electrode, and microfluidic devices too—providing useful insights on point-of-care healthcare devices.

### 3.1. Optical Biosensing Devices

Optical biosensing devices have been developed to offer a simple and rapid approach for sensing biological analytes. This method has been classified into, mainly, four techniques: colorimetric, fluorescent, surface plasmon resonance (SPR), and surface-enhanced Raman scattering methods, which are combined with magnetic particles in integrated devices; these were reported in proof-of-concept studies. Here, we discuss how the magnetic particles can be adapted to the optical sensing systems.

#### 3.1.1. Colorimetric Biosensing Devices

Colorimetric biosensing techniques are to transform the detection of biological elements into measurable color changes. Additionally, they are inexpensive and only require simple detection equipment [36,37]. For various colorimetric devices for analysis, lateral flow tests (LFA) and paper devices are the main methods which are commonly associated with enzyme-linked immunosorbent assay (ELISA) to identify targets [38]. As mentioned earlier, these devices combined with MNPs have benefits such as sensitivity, specificity, speed, and inexpensive platforms [39]. Moreover, due to the small testing sample volume, vulnerable individuals being exposed to dangerous chemicals is not an issue. Lastly, colorimetric tests are primarily tested with digital images, leading to an objective automated analysis by smartphone or camera [40,41]. Table 2 summarizes magnetic-materials-based colorimetric devices developed and employed for the quantification of biological targets.

Recently, Russell et al. depicted a sensitive colorimetric assay for the detection sepsis biomarkers in whole blood on filter papers [50]. Biotinylated capture antibodies and avidin-labeled particles were used to remove non-specific interactions with target PCT. It was followed by the motion-to-color transduction process and further amplification via Janus nanoparticles. Finally, these color changes are then read and quantified by customized smartphone apps. The assay depicted high sensitivity (limit of detection (LOD) = 2 ng·mL^−1^) in the dynamic linear range of 1–20 ng mL^−1^ within 13 min and potential application in whole blood without preparation. And another example is from Li’s group, who published a rapid and effective method for a *L. monocytogenes* gene; it is a major infectious pathogen that threatens public health worldwide [51]. In this study, in the strong interaction between streptavidin and biotin, the immunomagnetic-streptavidin is effectively attached to *L. monocytogenes* cells for high molecular identification and catalytic activity. The genomic DNA of *L. monocytogenes* was extracted, and PCR was performed to create single-strand DNA amplifiers (ssDNA). Finally, through the nucleic acid lateral flow (NALF) biosensor, ssDNA amplifiers were detected by the naked eye. The LOD and the linear range for *L. monocytogenes* were 3.5 × 10^3^ colony forming unit (CFU) mL^−1^ and 10^0^ to 10^7^ CFU mL^−1^, respectively.

#### 3.1.2. Fluorescent Biosensing Devices

Fluorescent biosensing devices are based on changes caused by analytes in the chemical and physical properties of fluorophores, including fluorescence intensity, lifetime, and anisotropy, in connection with the process of charge transfer or power transmission process [52,53]. In addition, it has been known as an effective method for multiple and highly sensitive detections of biological targets in molecular and clinical diagnosis to monitor disease progression and drug/therapy method response to diagnose images and image-guided surgery [54,55,56,57]. The use of magnetic particles enables the high washing efficiency of the target separation step to rapidly remove the unwanted components, resulting in the enhancement of the limit of detection. Based on those advantages, widely used fluorescence-based technologies for multiple/highly sensitive detections using the magnetic beads, such as Luminex xMAP [58,59,60,61], magnetic modulation biosensing (MMB) [62], Quanterix Simoa [63,64] and magnetically-modulated optical nanoprobes (MagMOONs) [65,66], have emerged. A recent study by Wang et al. pointed out a microfluidic biosensing device modification platform for *Salmonella typhimurium* measurement using fluorescent labeling and video processing on smartphones. Magnetic nanoparticles (MNPs) were modified with monoclonal antibodies against *Salmonella typhimurium* and then reacted with modified fluorescence microscopy (FMS) with polyclonal antibodies against *Salmonella typhimurium* to form a FMS-*Salmonella* complex. The structure of a smartphone-based fluorescent microscope system has been used with LED light sources to excite fluorescence to track the fluorescence points and the number of fluorescent bacteria calculated via real-time video processing within 2 h. The detection limit (LOD) and linear range of *Salmonella typhimurium* were 58 CFU mL^−1^ and 1.4 × 10^2^ to 1.4 × 10^6^ CFU mL^−1^, respectively. The existence of *Listeri monocytogenes*, *Escherichia coli O157: H7,* and *Vibrio parahaemolyticus* in samples did not interfere with the detection of *Salmonella typhimurium* [67].

Other fluorescence methods to detect biological targets include the use of composite materials in which magnetic nanoparticles serve as the main material to facilitate higher detection capacity. To perform this method, several studies have tried to incorporate magnetic nanoparticles known to be capable of separating samples into a specific target of interest. In 2019, Zhang et al. developed fluorescent, magnetic, spore-based (spore@Fe_3_O_4_@CDs) microrobots (FMSMs) for detecting toxins secreted by *Clostridium difficile* bacteria. Based on fluorescence property, the detection limit of this platform for *Clostridium difficile* was 2.13 ng mL^−1^ and the linear range was 0.38–17.80 ng mL^−1^ [68]. Therefore, owing to the selectivity of magnetic materials, this technique could be an effective method for various biological targets. Burg et al. also reported a cluster of magnetic particles based on fluorescence. Since the fluorescence is based on a cluster of magnetic beads with an active conic tip, a cluster of magnetic beads provides far greater fluorescent signals than single-particles. Human interleukin-8 was detected by fluorescent devices with a CMOS camera. The detection limit was 0.1 ng L^−1^ [69].

#### 3.1.3. Surface Plasmon Resonance Biosensing Devices

Surface plasmon resonance (SPR) sensing techniques are based on photonic technology, electronics, and nanotechnology for label-free optical sensing technology, which allows for direct refractive index changes and real-time sensor surfaces, providing excellent sensitivity due to magnified electric fields. The rapid and widespread advancement of SPR technology has been done by using magnetic materials for the intensity of the sensitivity of reflected light at a specific angle called the resonant angle [70]. The changing color of the solution can be observed due to the change in reflectance angle or wavelength against time in SPR system [71,72]. Immunoassays with SPR devices have been successfully developed to detect the extracellular vesicles by using antibodies against CD81 [73]. Meanwhile, Reiner’s group has utilized magnetic nanoparticles to develop the novel method of SPR combined with fluorescence, which resulted in 2.4-fold higher fluorescence than just an SPR detection channel only. In addition, a smartphone integrated compact system has been developed to detect different concentrations of antibodies under the nM range sensitivity, and the device has a resolution of 7.4 × 10^−5^ refractive index unit (RIU) and weight of 40 g [74].

#### 3.1.4. Surface-Enhanced Raman Scattering Biosensing Devices

**Figure 2:**
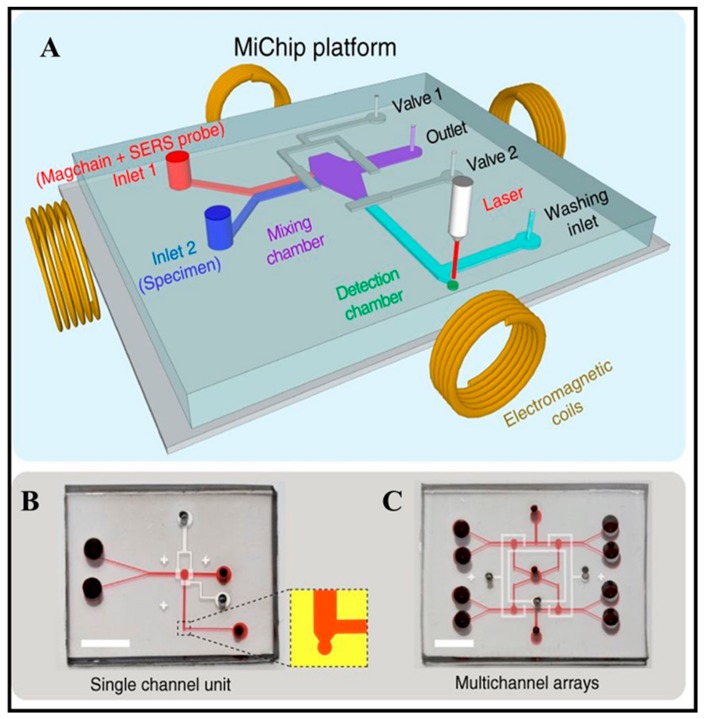
(**A**) Schematic illustration of the Magchain-integrated microchip (MiChip) assay platform: with (**B**) a single-channel unit and (**C**) multiple-channel arrays. Reproduced with permission from Xiong et al. [78].

**Figure 3:**
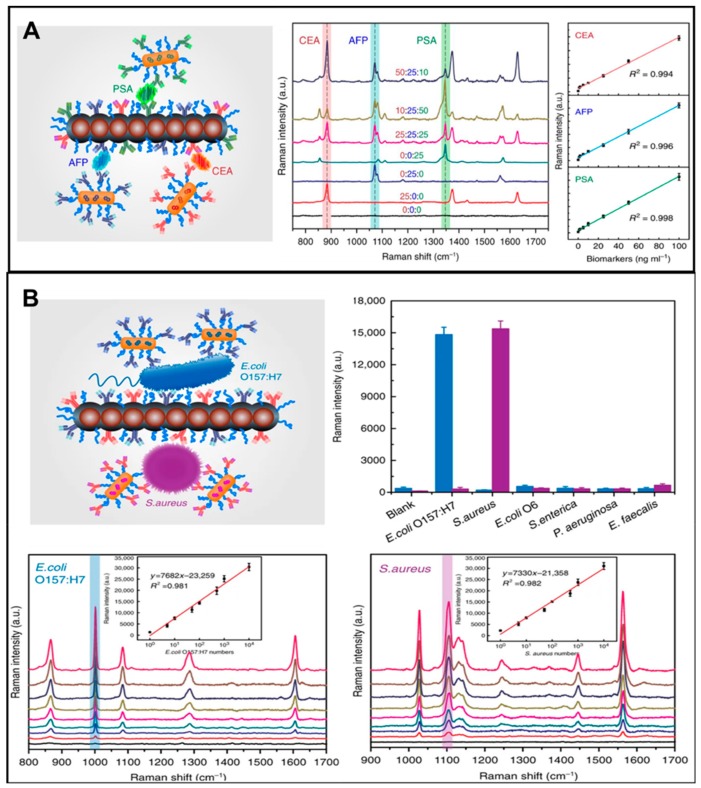
(**A**) Schematic, surface-enhanced Raman scattering (SERS) performance, and corresponding calibration curve with a mixture biomarkers (PSA, AFP, and CEA) at different concentrations from 0 to 100 ng mL^−1^; (**B**) schematic illustration of in-situ screening of detection *of Escherichia coli* O157:H7 and *Staphylococcus aureus* based on an MiChip assay with various concentrations (ranging from 0 to 104 CFU μL^−1^) and the results of saliva spiked samples in MiChip assay. Reproduced with permission from Xiong et al. [78].

Surface-enhanced Raman scattering (SERS) was founded in the 1970s by M. Fleischmannand et al. and can generate the spectral signatures of various biological analytes with its high sensitivity, specificity, and speed. Especially, SERS has intense penetration in complex biological sample matrices, such as in blood, and in pork samples [75,76,77]. For example, Xiong et al. reported the magnetic particle integrated microfluidic chip applied in SPR for a simple, rapid, and highly sensitive detection of multi cancer biomarkers (prostate specific antigen (PSA), alpha-fetoprotein (AFP), carcinoembryonic antigen (CEA), cancer antigen (CA) 125, and CA19-9) with the detection limit of PSA down to 0.2 pg mL^−1^ and bacterial species (*E. coli* O157: H7 *and S. aureus*) in 1 μL of body fluids within 8 min (Figure 2 and Figure 3) [78]. Zhang’s group also demonstrated that *S. typhimurium* and *S. aureus* were detected by using aptamer probes and magnetic gold nanoparticles with enhanced Raman intensity under the SERS intensity at 1582 cm^−1^ in the range of 10^2^–10^7^ CFU mL^−1^. The limits of detection are 35 CFU mL^−1^ for S. aureus and 15 CFU mL^−1^ for *S. typhimurium*, respectively [79].

### 3.2. Electrochemical Biosensing Devices

Electrochemical biosensing techniques require the understanding of the electrical properties of the detection method, such as its potential, current, capacitance, and impedance, to measure the electric current generated by the oxidation and reduction reactions of analytical targets. They are directly proportional to the concentrations of analyte in complex biological samples [80,81,82]. We can categorize the system of the electrochemical detection in biological samples into four techniques: potentiometric, conductometric, amperometric, and impedance biosensing devices (see Table 3). The development of electrochemical equipment, when combined with magnetic materials, has led to great potential for commercialization of biosensing devices.

#### 3.2.1. Potentiometric Biosensing Devices

Potentiometry is an engaging instrument for many practical applications, because it allows the measurement of a wide spectrum of ions, and it applies mobile and reasonable price devices in numerous medical, industrial, and environmental tests [36,83,84]. The potentiometric transduction was first reported in 1969, in which an enzyme based sensor was used to detect urea [85]. It involves measuring the potential difference between the working and reference electrodes in order to generate different potentials from analysis when providing a constant voltage. In addition, there is no significant current flow between them [86]. Potentiometry usually has a large dynamic range due to the signal proportional to the logarithm of the ionic activity. In addition, they have a short response time, in the order of seconds, making them suitable for process control and enabling high sample throughput in flow analysis. Moreover, their small size, reasonably economical production values, low power expenditure, and portability, make them more proper for diverse purposes [87,88]. Liu et al. presented a specific single-cell detection device that is based on target cells that are conjugated with magnetic beads for the magnetic bead assay and two micro Coulter counters for potentiometric detection of human umbilical vein endothelial cells (HUVECs) [89]. The HUVECs’ obviously have a much longer transit time distribution than the non-target rat adipose-derived stem cells (rASCs). They also reported that the various cell ratios (2%, 5%, 10%, 30%, and 50%) of HUVECs which can be detected in situ were accurately identified.

#### 3.2.2. Conductometric Biosensing Devices

In this type of device, analytical information is obtained by measuring the electrical conductivity, due to the charge transmission of cations and anions under the action of an external electric field. In other words, the measurement is based on an electroanalytical method that involves measuring conductivity separated by a specific distance or environment, such as nanowires. Conductivity measurement is based on the alternating current (AC) power supply to apply across the electrode using an Ohmmeter. The main advantages of the instrumentation are that they do not require a reference electrode, are inexpensive, have the ability to minimize the direct electrical response, are fast, and are single-use biological devices [90,91,92]. In this study, urease based magnetic beads were modified with graphene oxide and nickel oxide to have conduction of interfacial electrons and high enzyme binding activity [93]. Upon the target outline in urea samples, the wireless measurement system and the microfluidic measurement system proceeded to separate testing assays. While using a microfluidic device, the developed biosensing device could measure urea at the sensitivity 5.582 mV (mg/dl)^−1^ with a linearity of 0.959 and high reliability.

#### 3.2.3. Amperometric Biosensing Devices

**Figure 4:**
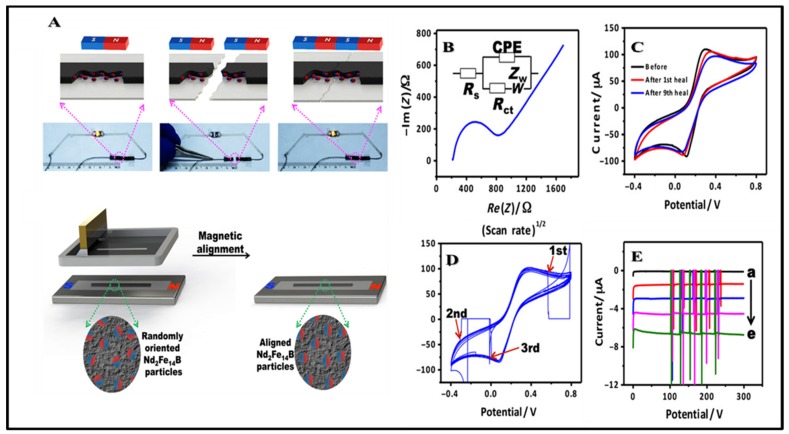
(**A**) Schematic diagram to illustrate the fabrication of self-healing principle and the manufacturing process; (**B**) Nyquist plot for self-healing printed electrodes; (**C**) cyclic voltammetry (CV) plots recorded for a self-healing trace; (**D**) real-time recovery of triple damage 3 mm wide; (**E**) amperometric response of the fabricated self-healing H_2_O_2_ in different concentrations from 0 to 20mM and under 1-mm-wide damage repetition. Reproduced with permission from [60].

Amperometry is the one of the most sensitive techniques to obtain high sensitivity in biosensor devices. This technique is based on the measurement of electric current as a function of the time due to the oxidation and reduction of an electrolyte in the biochemical reaction, largely depending on the concentration of the analyte of constant potential. Because most analytes (proteins) cannot essentially act as redox partners in the electrochemical reaction, electrons are also not transferred from the analyte to the working electrode or to the analyte from the electrode. To overcome that problem, a labeling electrochemical test is required for the electrochemical reaction of the analyte at the electrode surfaces. Horseradish peroxidase (HRP) and alkaline phosphatase (ALP) are the most commonly used as labels to catalyze the reaction of substrates to form electroactive products [94,95]. Bandodkar’s group recently represented a rapid, sensitive, and reasonable price in printed electrochemical devices for the detection of H_2_O_2_ at self-healing electrodes [96]. As illustrated in Figure 4, a Nd_2_Fe_14_B microparticle (NMP)–loaded graphitic inks were used to improve the self-healing ability with highly repeats recovery. It was followed by amperometric quantification of H_2_O_2_, with repeat five times for each H_2_O_2_ concentration at the same location. This assay produced a new self-healing notion, and the inherent application can also be applied in the wearable device based electrical circuits.

#### 3.2.4. Impedimetric Biosensing Devices

In electrochemical impedance spectroscopy (EIS), there are interference characteristics of surface-changing electrodes and mechanisms of charge transfer and ion transport in the electrolytic interface, which have recently become widespread tools for bioreceptors. This method is known as an oscilloscope scan with a frequency of electrical sweep of the immunoassay system, within 10 kHz and 10 mHz, with these parameters being resistance (R), resistance transfer charge (Rct) between solution and electrode surface, Warburg element (Zw), and double-layer capacitance (Cdl) [97,98]. The sample impedance is calculated as the ratio of voltage to current with both amplitude and phase: a complex number. EIS may have the strongest advantage of lower concentration detection (nano unit and pico unit) and is capable of testing for unlabeled detection [99,100]. In one of the studies by Wang et al., thrombin was electrochemically tracked in biological samples in serum using a microfluidic system and magnetic separation functionalized with the detection of protein. The probe thrombin-aptamer was coated magnetic beads to capture and separate thrombin target. After the target was injected into the microfluidic flow cell, the values of impedance changed, owing to the fact that the concentration of thrombin is proportional to the charges on the thrombin surface. This microfluidic device submitted a notable, rapid, specific, and sensitive method, with a detection limit of 0.01 nM and a linear range from 0.1 nM to 10 nM [101]. Another study was conducted by Kongsuphol’s team to detect tumor necrosis factor (TNF-α) by using a magnetic antibody biosensor-based biosensor platform [102]. Abundant protein sources are drained from the serum by using magnetic particles in combination with albumin and IgG antibodies. TNF-α is then captured by TNF-α antibodies conjugated with magnetic particles. The conjugated TNF-α was eluted from magnetic particles and measured using EIS technique which combined a structured gold microelectrode array (CSGM) to detect ultrasensitive 1 pg mL^−1^ with undiluted human serum and linearity from 1 to 1000 pg mL^−1^. Both published methods are designed to be sensitive, simple, and label-free platforms. As shown in Table 3, we summarized the strategies for electrochemical biosensor using magnetic particles. 

## 4. Magnetic Phenomena-Based Bioassays

In this section, we will discuss magnetic phenomena used in biosensing platforms. Magnetic materials are basically composed of metals, such as Fe, Co, or Ni, or metal oxides. Since they provide various advantages, such as larger surface area, specific controllability through a magnetic field, and functional alterations through surface modifications, they are often used to fulfill requirements for developing advanced forms of biosensing as ways of separating and pre-treatment of samples or used as detection probes for enhancing measured signals. Moreover, magnetic particles have been employed in various magnetic phenomena-based bioassays. This review will categorize recent magnetic nanoparticle-based assays into giant magnetoresistance (GMR), magnetic tunnel junction (MTJ), magnetic particle spectroscopy (MPS), and the nuclear magnetic resonance (NMR)-based assay.

### 4.1. GMR-Based Bioassay

**Figure 5:**
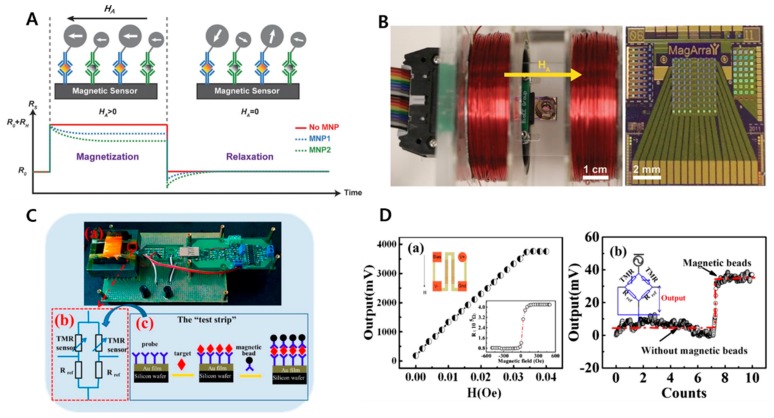
(**A**) Illustration of giant magnetoresistance (GMR) biosensors for time-domain magnetorelaxometry with the corresponding resistance signals in response to the external magnetic field; (**B**) simplified measurement setup with electromagnet and sensor array and GMR sensor array; (**C**) the Wheatstone bridge based on tunnel magnetoresistance (TMR) sensors and the sandwich assay on a test strip; (**D**) the results of the system under different magnetic fields, and the real-time response of the system when applying the magnetic beads. Inset: magnetic field dependence on the resistance of a TMR sensor. Reproduced with permission from [122,127].

Giant magnetoresistance (GMR) biosensor employs the basic phenomenon that occurs in magnetic materials including nanoparticles, multilayered thin films, and permanent magnets combined with bioreceptors. This appears alteration of magnetization in so called “free-layer” which consists GMR sensor platform, leading to the change of the resistance of GMR sensors [112] The detection takes place through measuring the signal intensity, which would be proportional to number of nanoparticles bound to the unit area [113]. It has been demonstrated that GMR sensors show highly sensitive and real-time signal readout. Furthermore, they can be produced with low cost which will be advantageous for mass production. [114,115,116] Through surface modification of MNPs, this platform can be widely used for detection of several biological targets as shown in Figure 5A,B. [113,117,118,119,120,121,122].

### 4.2. MTJ-Based Bioassay

Magnetic tunnel junction (MTJ) sensors, also one of the magneto resistive sensors, often called as tunnel magnetoresistance (TMR) sensor require low magnetic field while still offering high sensitivity because they possess higher ratio of magnetoresistance compared with GMR. Furthermore, there are various important qualities of TMR sensor platform such as convenience, less demand of sample quantity, wider conditions for choices of working temperature and voltage [123,124]. Furthermore, this feature can lead to fabrication of power-saving sensors, strengthening the merits of producing economic and eco-friendly bioassay devices [125]. TMR is caused by spinning coming from electron tunneling. Several studies have done for detecting bio recognition elements. For instance, Xiao’s team detected magnetic nanoparticles conjugated with DNA on MgO-based sensor surface [126]. To detect *E. coli* O157:H7 while using test strips, capture antibody-conjugated magnetic beads were employed. Signals were measured based on a potential change caused by difference of magnetic field fluctuation pattern due to the contrast between existence and absence of target binding to antibody-conjugated MNPs [127] (see Figure 5C,D). Mu et al. also conducted a study of detecting ricin on a test strip with magnetic field generating platform constituting of vertical and horizontal coils [128].

### 4.3. MPS-Based Bioassay

Magnetic particle spectroscopy (MPS), also known as magnetization response spectroscopy, is a newly rising technique that applies a sinusoidal magnetic field applied to superparamagnetic iron oxide nanoparticles and observes their periodical saturation of magnetization in response to the magnetic field [129,130]. MPS signal is only observed from magnetic nanoparticles, rather than iron in blood or biological tissue, which makes it more accurate to quantify MNPs unaffected by other undesired factors [131]. Combined with Brownian relaxation methods, biotin-coated MNPs captured streptavidin successfully, which also showed sensitivity of detecting streptavidin at as low a concentration as 75nM. The demonstrated study adopted the changes in MPS patterns that occur when MNPs are bound to specific target, while diminishing the need of washing process [132].

### 4.4. NMR-Based Bioassay

**Figure 6:**
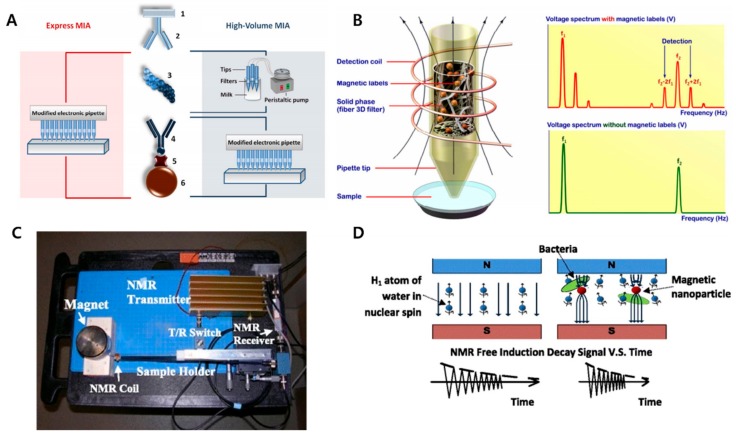
(**A**) Illustration of magnetic immunosandwich on 3D fiber filters used as a solid phase: 1— filter surface, 2—capture antibody, 3—antigen, 4—biotinylated tracer antibody, 5—streptavidin, 6—magnetic nanoparticles; (**B**) detection of superparamagnetic nanolabels by their non-linear response at combinatorial frequencies from the whole volume of 3D solid phase located inside a pipette tip; (**C**) portable nuclear magnetic resonance biosensor; (**D**) assay for the highly sensitive and rapid detection of foodborne bacteria in complex matrices. Reproduced with permission from [130,134].

Nuclear magnetic resonance is a physical event that occurs when a certain atomic nucleus is placed in magnetic field to absorb electromagnetic radiation and released again. Through numerous investigations of this event, one can apply this event to analyze the magnetic characteristics of nuclei and develop the information to detect bio-molecules as well [133]. Due to the facts that this signal can pass through samples without destructing them and sample preparation time is not necessary, NMR methods are adequate to analyze biological samples, and to save up time for analysis. With various modifications to improve this technique, fast and accurate detection with small sample volume has been made possible, also enabling mass production of handy-sized, easy-of-use sensor platform with low cost [134,135]. Addition of magnetic nanoparticles to the platforms can guarantee higher sensitivity and shorten the testing time. To detect prostate-specific antigen, nitrocellulose membrane-based test strips were designed and measured by a portable NMR relaxometer [136]. Many other research groups attempted to adopt this technique to detect several biological components such as food borne bacteria [137,138,139,140] (See Figure 6).

## 5. Concluding Remarks and Future Perspectives

Recent advances in magnetic particles have been focusing on employing various biomedical applications to achieve highly sensitive and rapid sensing performance. Magnetic particle-based target-molecule separation processes for sample preparation have been developed and reported by multiple research groups. For point-of-care biosensing devices, various magnetic particles were easily applied for the enhanced performances of biosensors with various techniques, such as the optical/electrochemical method combined with a microfluidic channel or LFA system, and also various magnetic phenomena-based bioassays, resulting in greatly simplifying extraction and detection procedures. In this review, the use of magnetic materials in the efficient isolations of various molecules and cells, and their successful use in many types of diagnostic biosensors, were summarized. These simple, rapid, and sensitive approaches may show promising developments in point-of-care and high-throughput biological devices for precise testing and analysis within a short time, greatly improving human health in the future.

## Figures and Tables

**Table 1:** Examples of proteins/peptides purified by magnetic techniques.

Purified Protein/Peptide	Source	Magnetic Carrier	Affinity Ligand	Further Details	Ref.
**Anti-DNA antibody**	Systemic lupus erythematosus patient plasma	Magnetic poly(2-hydroxyethyl-methacrylate) beads	DNA	Desorption with 1 M NBaSCN solution	[25]
**Immunoglobulin G**	Blood serum	Carboxyl-terminated magnetic particles	MproteinAG (fused-Fc-binding protein staphylococcal A and G )	-	[26]
**IgE antibodies**	Allergic patients sera	Magnetoliposomes	Antigenic proteins	-	[27]
**CUG binding proteins (RNA binding protein1)**	Human myoblasts or fibroblasts	Dynabeads M-280 streptavidin	Biotinylated(CUG)_10_	Elution with 1 M NaCl	[28]
**DNA-binding proteins**	HeLa nuclear extracts	Dynabeads M-280 streptavidin	Biotin-labelled DNA fragment	Elution with 2 M NaCl	[29]
**Pigpen protein**	Endothelial cells	Magnetic streptavidin beads	Biotinylated aptamer	Elution with 1 M NaCl	[30]
**Transcription proteins**	Human myeloid cells	Dynabeads M-280 streptavidin	Biotinylated serum inducible element	Elution with high salt buffer	[31]
**Glycated heamoglobin**	Human blood	Magnetic poly(vinyl alcohol) beads	m-Aminophenyl-boronic acid	Elution with sorbitol	[32]
**(His)_6_-Ala-Tyr-Gly**	Synthetic peptide	Dynabeads M-280 tosylactivated	Aminocaproic nitrilotriacetic acid charged with Ni^+^	Elution with imidazole solution	[33]

**Table 2:** Summary of the strategies used for magnetic-biomaterials-based colorimetric devices.

Types of Detection Platform	Target	Magnetic Biomaterial	Linear Range	Limit of Detection (LOD)	Advantageousness	Detection Time	Ref.
Paper-based analytical device (PAD)	Bacteria *(S. typhimurium)*	Magnetic beads	10^2^ to 10^8^ colony forming unit (CFU) mL^−1^	10^2^ CFU mL^−1^	Simple, rapid and sensitive without pre-enrichment sample	90 min	[42]
Lateral-flow immunochro-matographic assays (LFIA)	Antigen (hCG)	Fe_3_O_4_−Pt/ core−shell nanoparticles	0 to 1 ng mL^−1^	0.039 ng mL^−1^	Sensitive more than conventional Au-LFIA	-	[43]
Lateral-flow immunochro-matographic assays (LFIA)	Bacteria *(B. anthracis)*	Super-paramagnetic particles	2.0 × 10^3^ to 1.0 × 10^6^ CFU mL^−1^	7.0 × 10^3^ CFU mL^−1^	Sensitive, specificity, cost and ease of operation	20 min	[44]
Paper-based MNP-gold sensing assays	Bacteria *(Escherichia coli O157:H7)*	Magnetic nanoparticles	1.21 × 10^1^ to 1.21 × 10^6^ CFU mL^−1^	12 CFU mL^−1^	Simple to perform, low cost, rapid without pre-enrichment sample	Less than 30s	[45]
Paper-based analytical devices	Antigen (PCT)	Janus particles	1 to 20 ng mL^−1^	2 ng mL^−1^	Not required any pre-enrichment sample	13 min	[46]
Lateral-flow immunochro-matographic assays (LFIA)	Antigen (CFB)	Magnetized Carbon Nanotube	5 to 100 ng mL^−1^	5 ng mL^−1^	Rapid and low-cost tool	30 min	[47]
Peroxide strip based assay analyzed by Smartphone	Bacteria *(S. typhimurium)*	Immunomagnetic nanoparticles	10^1^ to 10^5^ CFU mL^−1^	1.6 × 10^1^ CFU mL^−1^	Simple, portable and low-cost method for rapid detection	45 min	[48]
Nucleic acid lateral flow (NALF) assay	Nucleic acid *(L.* *monocytogenes)*	Immunomagnetic nanoparticles	10^0^ to 10^7^ CFU mL^−1^	3.5 × 10^3^ CFU mL^−1^	Excellent viable capability for viable *L. monocytogenes*	6 h	[49]

**Table 3:** Summary of the strategies used for magnetic biomaterials based electrochemical biosensing devices.

Device Concepts	Target	Magnetic Biomaterial	Detection Range	LOD	Advantageousness	Detection Time	Detection Method	Ref.
Microfluidic device	Human umbilical vein endothelial cells (HUVECs)	Magnetic beads	13 to 100 cell µL^−1^	-	Simple device and easy operation	-	Potentio-metry	[89]
Wireless measurement system and microfluidic measurement system	Urea	Magnetic beads	0 to 50 mV (mg/dl)	4.780 mV (mg/dl) ^−1^ for wireless measurement and 5.582 mV (mg/dl) ^−1^ for microfluidic measurement	Reliability measurement	-	Conducto-metry	[93]
An immobilization- free interdigitated array microelectrode	Bacteria *(L* *monocytogenes)*	Immunomagnetic nanoparticles	3.0 × 10^1^ to 3.0 × 10^4^ CFU mL^−1^	300 CFU mL^−1^	Simple, low-cost and sensitive method for rapid screening	-	Impedi-metry	[103]
Digital microfluidics - Nanostructured microelectrodes (DMF-NME) device	Rubella virus (RV)	Magnetic beads	0 to 250 IU mL^−1^	0.07 IU mL^−1^	Sensitive device with the small size	30 min	Ampero-metry	[104]
Flexible film -based devices	Viruses (HIV)	Magnetic beads	10^6^ to 10^8^ copies mL^−1^	10^6^ copies mL^−1^	Sensitive, robust, portable, and inexpensive device	-	Impedi-metry	[105]
Screen-printed car- bon electrodes (SPdCEs)	Antigen (IL-8 protein) and IL-8 mRNA	Magnetic beads	-	0.21 nM for IL-8 mRNA and 72.4 pg mL^−1^ for IL-8 protein	Detectable in undiluted saliva samples and confirm test with commercial ELISA Kit	5 h	Ampero-metry	[106]
Screen-printed interdigitated electrode (SPIE)	Bacteria *(L* *monocytogenes)*	Immuno-magnetic nanoparticles	-	1.6 x 10^3^ CFU mL^−1^	Simple, low cost and good specificity	3 h	Impedi-metry	[107]
								
Screen Printed Carbon Electrodes (SPCEs)	Antigen (TPM)	Magnetic particles	0 to 218.7 ng mL^−1^	47 pg mL^−1^	High sensitivity and specificity	Under 3 h	Ampero-metry	[108]
Screen Printed Carbon Electrodes (SPCEs)	Nucleic acid (Sola l 7)	Magnetic beads	-	0.2 pM	Highly sensitive without pre-amplification sample, simple handling, low cost and safety	90 min	Ampero-metry	[109]
Screen Printed Carbon Electrodes (SPCEs)	Nucleic acid *(Ostreopsis* cf. *ovata)*	Maleimide-coated magnetic beads	-	9 pg µL^−1^	Sensitivity, specificity, storage stability and good correlation with other molecular methods	-	Ampero-metry	[110]
Paper microfluidics on screen-printed electrodes	Antigen (MMP-9)	Magnetic beads	30 pg mL^−1^ and 2 ng mL^−1^	0.01 ng mL^−1^	Simplify manipulation, providing fast, simple and sensitive assay formats	10 min	Ampero-metry	[111]
